# Human CRL4^DDB2^ ubiquitin ligase preferentially regulates post-repair chromatin restoration of H3K56Ac through recruitment of histone chaperon CAF-1

**DOI:** 10.18632/oncotarget.21869

**Published:** 2017-10-17

**Authors:** Qianzheng Zhu, Shengcai Wei, Nidhi Sharma, Gulzar Wani, Jinshan He, Altaf A. Wani

**Affiliations:** ^1^ Department of Radiology, The Ohio State University, Columbus, 43210, OH; ^2^ Department of Molecular and Cellular Biochemistry, The Ohio State University, Columbus, 43210, OH; ^3^ James Cancer Hospital and Solove Research Institute, The Ohio State University, Columbus, 43210, OH

**Keywords:** acetylated histone H3 lysine 56 (H3K56Ac), chromatin assembly, damaged DNA binding protein 2 (DDB2), CRL4^DDB2^ ubiquitin ligase, CUL4A and CAF-1

## Abstract

Acetylated histone H3 lysine 56 (H3K56Ac) diminishes in response to DNA damage but is restored following DNA repair. Here, we report that CRL4^DDB2^ ubiquitin ligase preferentially regulates post-repair chromatin restoration of H3K56Ac through recruitment of histone chaperon CAF-1. We show that H3K56Ac accumulates at DNA damage sites. The restoration of H3K56Ac but not H3K27Ac, H3K18Ac and H3K14Ac depends on CAF-1 function, whereas all these acetylations are mediated by CBP/p300. The CRL4^DDB2^ components, DDB1, DDB2 and CUL4A, are also required for maintaining the H3K56Ac and H3K9Ac level in chromatin, and for restoring H3K56Ac following induction of DNA photolesions and strand breaks. Depletion of CUL4A decreases the recruitment of CAF-1 p60 and p150 to ultraviolet radiation- and phleomycin-induced DNA damage. Neddylation inhibition renders CRL4^DDB2^ inactive, decreases H3K56Ac level, diminishes CAF-1 recruitment and prevents H3K56Ac restoration. Mutation in the PIP box of DDB2 compromises its capability to elevate the H3K56Ac level but does not affect XPC ubiquitination. These results demonstrated a function of CRL4^DDB2^ in differential regulation of histone acetylation in response to DNA damage, suggesting a novel role of CRL4^DDB2^ in repair-driven chromatin assembly.

## INTRODUCTION

In eukaryotes, the genome is organized into chromatin that carries both genetic and epigenetic information, which govern the fundamental functions of living cells. The basic unit of chromatin is nucleosome [1], consisting of two copies of each histone H2A, H2B, H3 and H4, as well as ∼146 bp of DNA wrapped nearly twice around the histone octamer. Chromatin structure acts as a barrier to several important DNA-templated processes, e.g., gene transcription, DNA replication and DNA repair. So, chromatin must be disassembled or undergo transient structural changes, allowing the normal DNA-templated processes in cells. Once these processes are culminated, chromatin ought to reassemble into the original state. Thus, chromatin restoration *via* chromatin assembly plays an important role not only in preserving epigenetic information following the execution of DNA-templated processes, but also in maintaining genomic stability upon repair of diverse DNA damage.

Chromatin restoration *via* chromatin assembly involves histone deposition onto replicated or repaired DNA by histone chaperons including chromatin assembly factor-1 (CAF-1) [2]. The CAF-1 complex contains three subunits, CAF-1 p150, p60 and p48, which are all required for *in vitro* nucleosome assembly function [3, 4]. CAF-1 interacts with proliferation cell nuclear antigen (PCNA) and deposits the replication-linked histone isoform H3.1 following DNA replication [5–7] and DNA repair [8–11]. Besides CAF-1, anti-silencing function 1 (Asf1) is also involved in chromatin assembly during both DNA replication [12, 13] and repair [14].

A previous genome-wide study in yeast has identified a genetic pathway which links histone acetylation and chromatin assembly to ubiquitination [15]. The Rtt101, Mms1 and Mms22 components of Rtt101^Mms1^ ubiquitin ligase function to maintain genome stability in the same pathway as yeast Asf1 and histone acetyltransferase Rtt109. It has been demonstrated that Asf1 forms a heterotrimeric complex with newly synthesized H3-H4, presents H3-H4 to Rtt109 for acetylation of histone 3 lysine 56 (H3K56) [15–19], and then transfers H3-H4 to CAF-1 for deposition. Recently, it was reported that Rtt101^Mms1^ ubiquitin ligase preferentially binds and ubiquitinates new acetylated H3K56 (H3K56Ac) and promotes the histone transfer [20]. However, the precise mechanism of histone transfer from Asf1 to CAF-1 remains unclear. In addition, mammalian ortholog of Rtt101^Mms1^ belongs to a DDB1-CUL4-Rbx1 ubiquitin ligase (CRL4) family [21, 22]. The CRL4 complex utilizes DDB1- and CUL4A-associated factors (DCAFs) as substrate receptors. The exact identity of DCAF for regulation of chromatin restoration *via* chromatin assembly is currently unknown.

While chromatin assembly results in the incorporation of H3K56Ac into chromatin during DNA synthesis, H3K56Ac in turn functions to drive chromatin assembly [23]. In mammals, histone acetyltransferases CBP/p300 are required for H3K56 acetylation in the presence and absence of DNA damage, whereas CAF-1 is required for the incorporation of H3K56Ac into chromatin [24, 25]. CBP/p300 acetylates both H3-H4 and Asf1-bound H3-H4 *in vitro,* but Asf1 is required for H3K56 acetylation *in vivo* [24]. In essence, histone acetyltransferases and histone chaperons cooperatively function to incorporate H3K56Ac into chromatin during DNA replication and DNA repair.

DNA repair-driven chromatin assembly is a late event in DNA damage response. The process is believed to restore damaged chromatin to its original states, or bookmark the once damaged chromatin with new post-translational histone modifications (PTMs). It is known that incorporated H3K56Ac in repaired chromatin signals the completion of DNA repair as well as termination of DNA damage induced checkpoint signaling activation [23]. Moreover, H3K56Ac also acts as one of PTMs, which is highly responsive in the early events of DNA damage signaling. Specifically, H3K56Ac rapidly diminishes in response to exposures to a variety of DNA damaging agents, including ultraviolet radiation (UVR), ionizing radiation (IR) and phleomycin [26]. Further studies have indicated that H3K56Ac is down-regulated by histone deacetylase (HDAC) 1 and 2 at DNA double strand breaks to promote DNA strand-break repair by non-homologous end-joining [27]. The unique biphasic decrease and increase of H3K56Ac mark in chromatin presumably reflects the changes in chromatin in response to DNA damage.

We previously found that H3K56Ac restoration during late time points following UVR requires functional nucleotide excision repair (NER) [28], and is dependent on Asf1a, which in turn is needed for dephosphorylation of phosphorylated histone H2A variant (γH2AX) and checkpoint recovery. We also found that the decrease in H3K56Ac in early phase of UVR response is mediated by combined action of HDAC1 and 2, and is regulated by DDB2 [29]. In present study, we examined the potential regulation of post-repair chromatin restoration of various histone acetylations, e.g., H3K56Ac, H3K9Ac, H3K18Ac, and H3K27ac and H3K14Ac, by CRL4^DDB2^, a member of CRL4 ubiquitin ligase family. Notably, DDB2 was originally identified as a component of damaged DNA binding activity [30]. DDB2 forms a heterodimer with DDB1 subunit and recognizes UVR-induced lesions and chemical-induced bulky DNA adducts. Further studies established that DDB2 and DDB1 are components of cullin 4 (CUL4)-based CRL4^DDB2^ ubiquitin ligase complex as mentioned above [31]. As an ubiquitin ligase, CRL4^DDB2^ mediates ubiquitination of several substrates including NER damage sensor XPC, histones H2A, H2B, H3 and H4, as well as the substrate receptor DDB2 itself [32–34]. Here, we identify a novel role of CRL4^DDB2^ in preferentially regulating chromatin restoration of H3K56Ac in a CAF-1 dependent a manner. We propose that genomic H3K56Ac restoration constitutes an integral part of repair-driven chromatin assembly.

## RESULTS

### CRL4^DDB2^ regulates H3K56Ac level and its restoration following DNA damage repair

**Figure 1:**
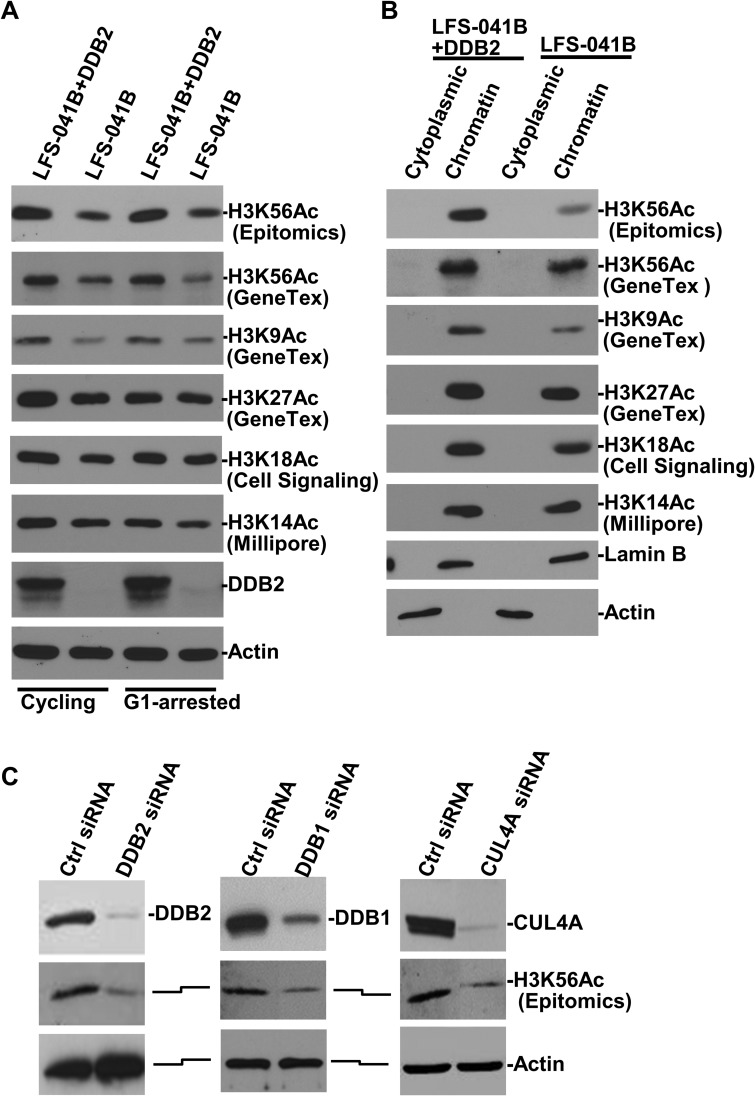
Depletion of CRL4^DDB2^ components DDB2, DDB1 and CUL4A decrease H3K56Ac levels (**A**) DDB2, H3K56Ac, H3K9Ac, H3K27Ac, H3K27Ac and H3K14 levels were examined in LFS-041B cell line and its derivative cell line with re-established DDB2 expression. (**B**) Cytoplasmic and chromatin fractions of LFS-041B and its derivative were obtained through a cellular protein fractionation protocol. Samples containing identical protein amounts from each fraction were examined by Western blotting for DDB2, H3K56Ac and other acetylated histone H3. Actin and Lamin B blots serve as the loading controls. (**C**) NHFs were transiently transfected with control (Ctrl) siRNA or siRNA targeting DDB2, DDB1 or CUL4A. Levels of DDB2, DDB1 and CUL4A as well as H3K56Ac were detected in whole cell Laemmli extracts by Western blotting.

Deficiency of the p53 function in Li-Fraumeni syndrome fibroblasts (LFS-041B cells) causes the suppression of DDB2 expression [35]. During establishment of stable cell lines of LFS-041B with enforced expression of DDB2, we found that restoration of DDB2 expression resulted in an elevated level of cellular of H3K56Ac. From the latest literature, we became aware that H3K56Ac antibody from Epitomics, used in our initial assessment, was reportedly exhibiting some cross-reactivity with H3K9Ac [36]. Therefore, to confirm the underlying phenomenon, we reexamined our results with a different H3K56Ac antibody, which as tested by the provider GeneTex using acetylated H3 peptides, does not harbor such cross-reactivity. As shown in Figure 1, H3K56Ac is elevated in both cycling and G1-arrested LFS-041B+DDB2 cells with enforced or corrected DDB2 expression, as compared with parental LFS-041B cells. H3K9Ac exhibited a similar elevation in G1-arrested LFS-041B+DDB2 cells albeit to a lesser extent. Observable differences in H3K27Ac, H3K18Ac and H3K14Ac were seen in cycling LFS-041B+DDB2 cells, but no substantial differences in H3K27Ac and H3K18Ac were seen in G1-arrested cells. The acetylated histones were exclusively detected in chromatin fractions (Figure 1B), indicating that these histone marks primarily resides in chromatin. Because DDB2 is part of CRL4^DDB2^ ubiquitin ligase functioning in NER, we further examined the phenomenon by individually knocking down DDB2, DDB1 and CUL4A, the three key components of CRL4^DDB2^ (Figure 1C). Compared with the control, depletion of DDB2, DDB1 or CUL4A all led to a coincident decrease in H3K56Ac, suggesting that these components function together in maintaining the constitutive H3K56Ac level in chromatin.

**Figure 2:**
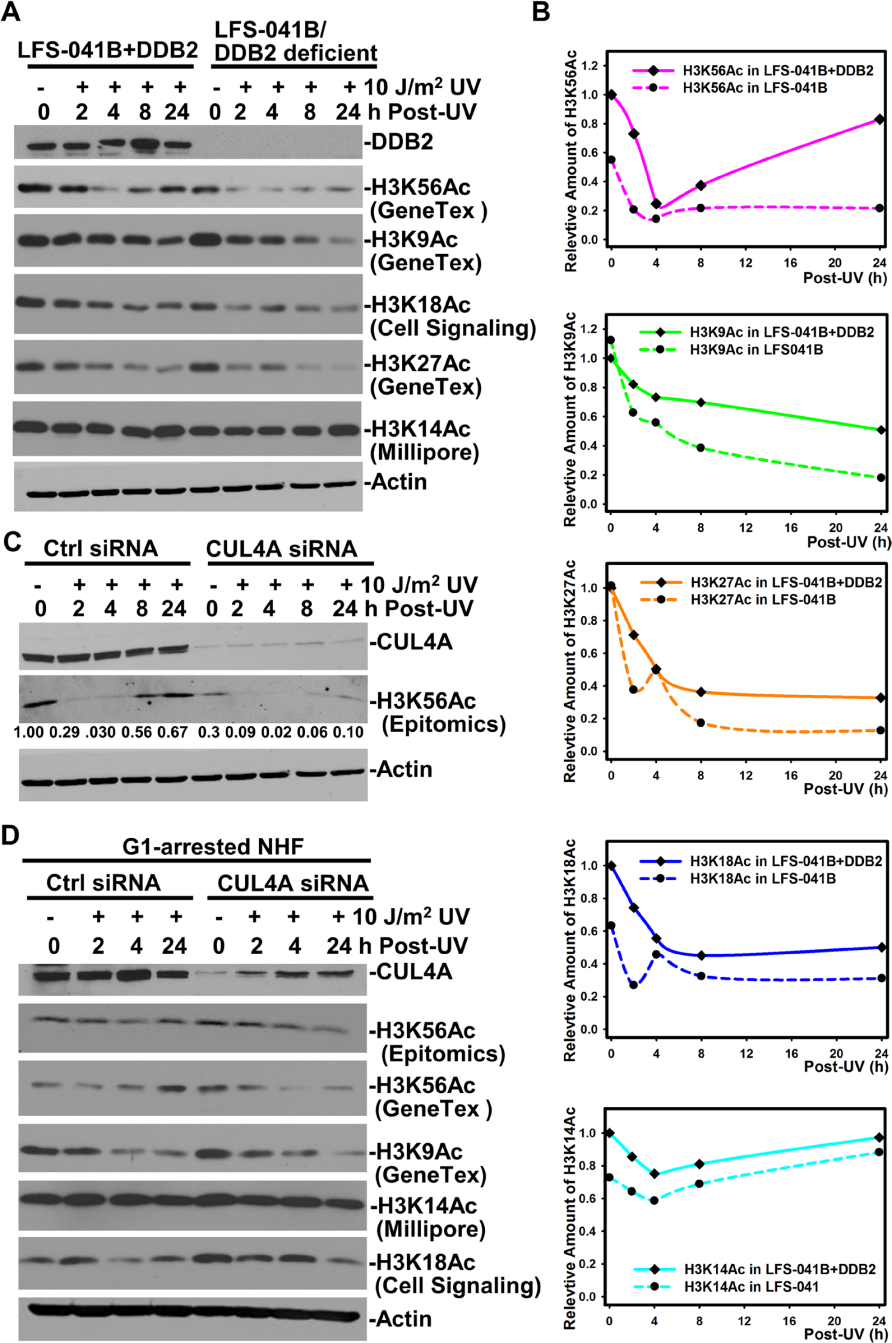
CRL4^DDB2^ components DDB2, DDB1 and CUL4A are required for restoring H3K56Ac post UVR treatment (**A**) LFS-041B and its derivative LFS-041B+DDB2 cells were exposed to UVR, maintained for indicated time periods and lysed by Laemmli lysis buffer. Samples of whole cell Laemmli extracts were analyzed by Western blotting for DDB2, H3K56Ac and other acetylated histone H3. (**B**) The blots from experiments in A were quantitated using ImageJ software and the relative protein levels were plotted against time course. The immunoblots of H3K56Ac with GeneTex anti-H3K56Ac antibody were used for ImageJ quantitation. (**C**) Western blotting analysis of CUL4A and H3K56Ac in control (Ctrl) siRNA and target-specific siRNA-transfected NHFs post UVR. (**D**) The siRNA-transfected NHFs were G1 arrested by serum starvation before UVR exposure and Western blotting analysis was the same as in B. Actin blots serve as the loading controls.

We next examined the DNA damage response patterns of H3K56Ac in cells with deficient DDB2 or the cells depleted of core CRL4 components DDB1 or CUL4A. Our earlier work has extensively established the biphasic UVR responsive pattern of H3K56Ac in HeLa and NHFs [28]. Specifically, H3K56Ac exhibits a typical dose-dependent decrease within the first 8 h followed by recovery from 8 to 48 h post-UVR time periods [28]. A similar pattern was observed for H3K56Ac in LFS-041B+DDB2 with enforced DDB2 expression, which keeps H3K56Ac at a relatively higher constitutive level (Figure 2A and 2B). As expected, H3K56Ac in LFS-041 cells exhibits H3K56Ac response pattern that begins with lower initial H3K56Ac level, and with much lower comeback at 24 h. Unlike H3K56Ac, however, H3K9Ac, H3K18Ac, H3K27Ac did not exhibit such a biphasic UVR responsive pattern in LFS-041B+DDB2 (Figure 2A and 2B). The decreases in their levels after UVR were apparent in parental LFS-041 cells. The H3K14Ac exhibited a very weak if any biphasic response, and the changes in H3K14Ac levels were not substantial regardless of DDB2 expression (Figure 2A and 2B). The quantitative assessment further documented that H3K56Ac recovery in LFS-041B cells is appreciably lower than its undamaged base level (Figure 2B). Thus, the data indicate that H3K56Ac recovery is preferentially regulated by DDB2 expression upon cellular UVR exposures.

We next examined the function of Cul4A, a key subunit of CRL4^DDB2^, in the regulation of H3K56Ac recovery upon UVR. In control siRNA-transfected NHFs, H3K56Ac follows the typical UVR-response pattern (Figure 2C). The pattern was more pronounced in siRNA-transfected NHFs than in LFS-041+DDB2 cells. This is presumably due to the enforced DDB2 at constantly high level in LFS-041B+DDB2 cells. More importantly, depletion of CUL4A by siRNA in NHFs not only decreased the constitutive level of H3K56Ac but also severely compromised the H3K56Ac recovery. Considering that the CUL4A depletion does not affect 6-4PP repair which occurs within the first 8 h [37] and that the CUL4A knockout enhances CPD repair [38], we argue that the effect of CUL4A depletion on H3K56Ac restoration cannot be due to failure of repairing photolesions.

To ensure that DNA replication does not confound our data interpretation, the siRNA-transfected NHFs were G1-arrested by serum starvation prior to applying UVR. The results showed that CUL4A depletion did not affect the H3K56Ac level without DNA damage in G1-arrested cells. More importantly, CUL4A depletion abolished the restoration of H3K56Ac in G1-arrested NHFs, but did not significantly affect the UVR response patterns of H3K9Ac, H3K18Ac and H3K14Ac (Figure 2D). Thus, CUL4A is functionally involved in maintaining H3K56Ac and also participated in H3K56Ac restoration following cellular UVR exposure. Taken together, we concluded that the CRL4^DDB2^ plays a role in chromatin recovery of epigenetic mark H3K56Ac in response to UV-induced DNA damage.

**Figure 3:**
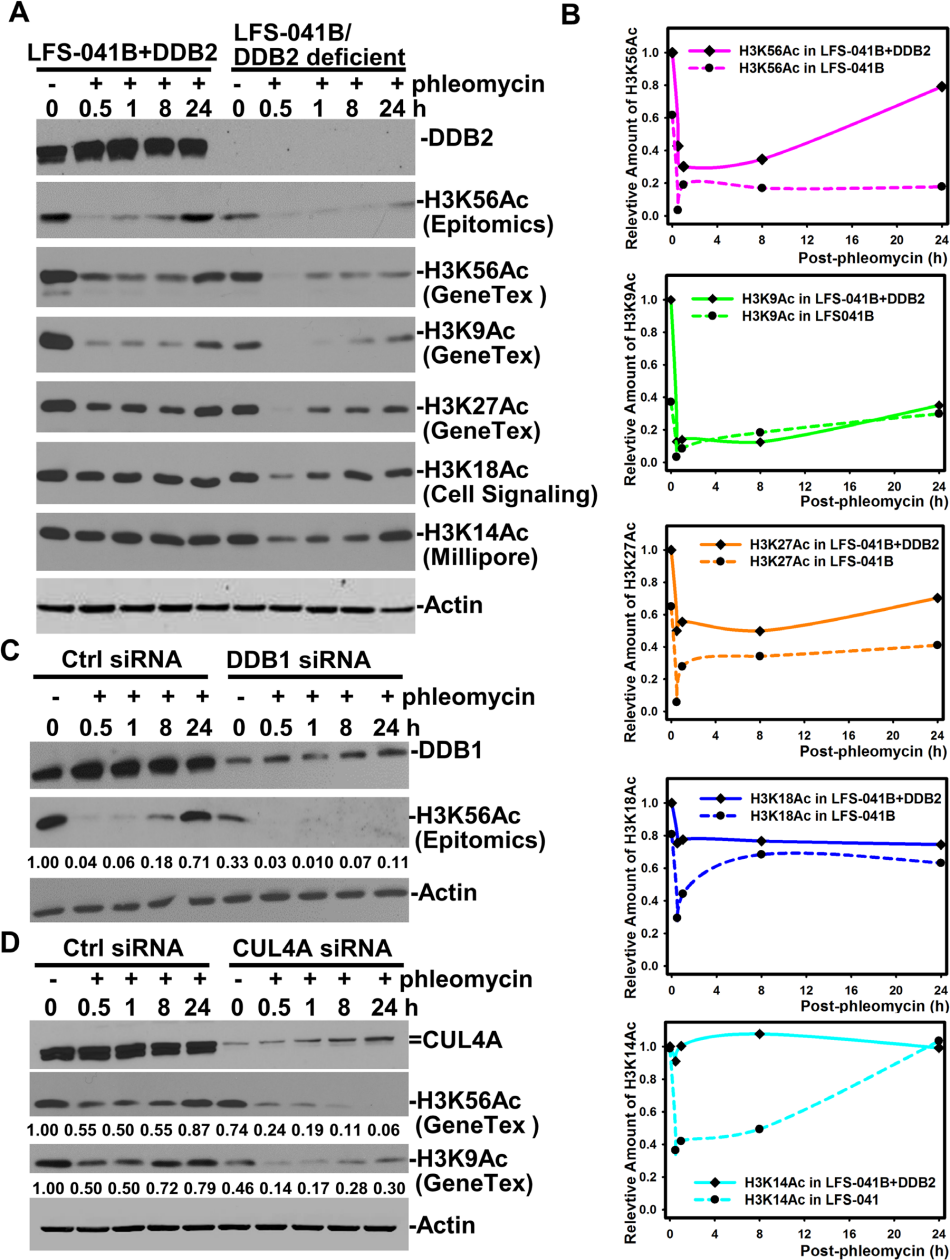
CRL4^DDB2^ components DDB2, DDB1 and CUL4A are required for restoring H3K56Ac post phleomycin treatment (**A**) LFS-041B and its derivative LFS-041B+DDB2 cells were treated with 60 μg/ml of phleomycin for 2 h. The treated cells were maintained in fresh medium for indicated time periods. Samples of whole cell Laemmli extracts were analyzed by Western blotting for DDB2 and H3K56Ac and other acetylated histone H3. (**B**) The blots from at experiments in A were quantitated using ImageJ software and the relative protein levels were plotted against time course. The immunoblots of H3K56Ac with GeneTex anti-H3K56Ac antibody were used for ImageJ quantitation. (**C**) Western blotting analysis of DDB1 and H3K56Ac in DDB1 siRNA-transfected NHFs upon phleomycin post-treatment. (**D**) Western blotting analysis of CUL4A and H3K56Ac in DDB1 siRNA-transfected NHFs upon phleomycin post-treatment. Actin blots serve as the loading controls.

To test if the regulation of H3K56Ac recovery is limited only to damage response following UVR exposures, we further examined the H3K56Ac recovery upon phleomycin-induced strand breaks. The H3K56Ac in LFS-041+DDB2 cells exhibited a sharp decline at 0.5 h and a clear renewal from 8 to 24 h after cellular phleomycin treatment (Figure 3A and 3B). At 24 h, H3K56Ac restored to ∼80% of the undamaged level. By contrast, the constitutive lower level of H3K56Ac in parental LFS-041B was diminished further following phleomycin treatment and exhibit ∼33% restoration of undamaged LFS-041B or ∼20% of undamaged LFS-041+DDB2 cells. Surprisingly, H3K9Ac in 041+DDB2 cells also exhibited a biphasic decrease and restoration after phleomycin treatment, despite that restoration of H3K9Ac only reaching to ∼40%. The H3K9Ac level was constitutively lower in LFS-041B as compared with that in LFS-041+DDB2 cells. The H3K9Ac in LFS-041B was fairly (∼ 50%) restored as compared to its level at 0 h time-point (Figure 3A). Additionally, in DDB2-overexpressing LFS-041+DDB2 cells, H3K27Ac, H3K18Ac and H3K14Ac did not exhibit a clear biphasic response to phleomycin treatment. However, in DDB2-deficient LFS-041 cells, phleomycin induced a clear biphasic response in levels of H3K27Ac, H3K18Ac and H3K14Ac, and, the levels of these histone modifications were restored to ∼70 to ∼80% of their levels at 0 h time-point (Figure 3B). We concluded that in case of phleomycin-induced strand breaks, DDB2 function is also preferentially required for chromatin restoration of H3K56Ac and, to lesser extent, for the partial restoration of H3K9Ac. We noticed that the H3K56Ac decrease observed upon UVR exposure occurs at a distinctly slower rate than that seen upon phleomycin treatment, suggesting that DNA strand-breaks are more potent triggers for H3K56Ac deacetylation. At this juncture, however, we do not understand the subtle differences between phleomycin-induced and UVR-induced response patterns of H3K9Ac, H3K27Ac, H3K18Ac and H3K14Ac in DDB2-deficent LFS-041B fibroblasts. The simplest explanation would be that the strand breaks trigger quicker and large scale changes in H3 acetylation than that by photolesions in these fibroblasts.

As compared with control siRNA, depletion of DDB1 (Figure 3C) or CUL4A (Figure 3D) by target-specific siRNA showed the similar damage-responsive pattern of H3K56Ac as that in DDB2-deficient LFS-041 cells (Figure 3A). In particular, the restoration of H3K56Ac in late hours after phleomycin treatment required both DDB1 and CUL4A function. In addition, the H3K9Ac pattern in CUL4A-depleted cells exhibited a limited partial restoration in DDB2-deficient LFS-041 cells (Figure 3A and 3D). Because phleomycin is known to cause DNA strand breaks rather than NER-repairable helix-distorting lesions recognized by DDB1-DDB2, we reasoned that CRL4^DDB2^ function in H3K56Ac restoration after phleomycin-induced DNA damage is not related to its function in NER.

### H3K56Ac restoration occurs specifically at DNA damage sites and is dependent on CAF-1 and histone acetyltransferase p300

**Figure 4:**
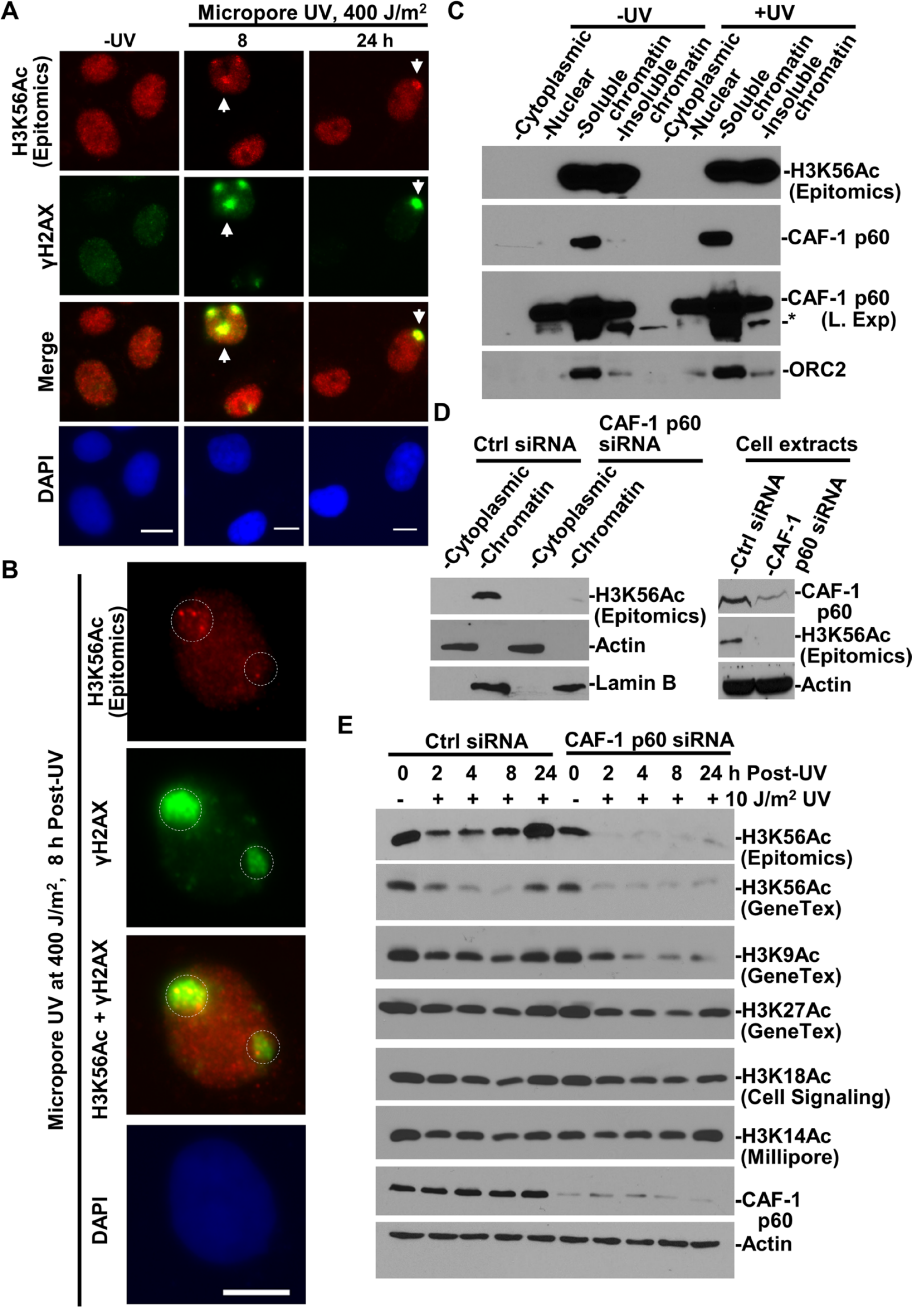
H3K56Ac accumulating at damage sites is incorporated into chromatin in CAF-1-dependent manner (**A**) H3K56Ac accumulates at DNA damage sites. H3K56Ac and γH2AX were visualized by immunofluorescence in HeLa cells exposed to UVR through a 5-μm isopore polycarbonate filter. Arrows indicate typical immunofluorescence focus of H3K56Ac or γH2AX. (**B**) A closer look at distinctive H3K56Ac foci at damage spots. Calibration bar is 10 μm. Dashed circles indicate the area of H3K56Ac and γH2AX foci. (**C**) Distribution of H3K56Ac, CAF-1 p60 in cellular protein fractions. HeLa cells were UV-irradiated at 20 J/m^2^, maintained for 1 h and subjected to cellular protein fractionation protocol. The cellular protein fractions containing the same amounts of proteins were analyzed by Western blotting. “L. Exp” indicates long exposure. ^*^ indicates a non-specific protein band. (**D**) H3K56Ac levels were examined in cytoplasmic and chromatin fractions from HeLa and CAF-1 p60-depleted HeLa cells. (**E**) H3K56Ac and other acetylated histone H3 levels were examined in NHFs and CAF-1 p60-depleted NHFs following UVR exposure. Actin blot serves as the loading controls.

DNA damage-induced H3K56Ac accumulation and restoration were detected at the global level by Western blotting and by immunofluorescence [24, 28]. We explored the possibility of visualizing H3K56Ac incorporation in the vicinity of UV-induced DNA damage sites. To do so, localized DNA damage was generated by micropore UVR through 5-μm isopore polycarbonate filters. Distinct UVR-induced fluorescent foci (UVRIF) of H3K56Ac, co-localizing with γH2AX, were detected in ∼10% of cells at 8 and 24 h following micropore UVR (Figure 4A). It may be noted that a relatively high micropore UVR dose is necessary to ensure that H3K56Ac UVRIF stand out from the native chromatin-embedded H3K56Ac background, which varies in cells due presumably to its uneven distribution on overall chromatinized genome. The appearance of H3K56Ac foci is seemingly due to H3K56ac overshoot during H3K56ac restoration after UVR [28] . A closer look at immunofluorescence indicated that a cluster of H3K56Ac foci with variable signal strength appear within the co-localizing γH2AX focal regions (Figure 4B), suggesting that H3K56Ac is not uniformly scattered in damaged chromatin. Thus, H3K56Ac restoration occurs locally at the damaged DNA regions.

Next, we examined the distribution of H3K56Ac in cellular protein fractions and investigated the relevance of CAF-1 function to H3K56Ac restoration after UVR-induced DNA damage. As mentioned, CAF-1 deposits histone H3-H4 following NER repair [8–11], but does not participate in early steps of NER. As expected, H3K56Ac was detected only in chromatin fractions, while CAF-1 p60 resides in chromatin fractions and to a lesser extent in nuclear/nucleoplasmic fraction regardless of DNA damage (Figure 4C). CAF-1 p60 depletion, through siRNA, effectively decreased the H3K56Ac levels in chromatin fraction (Figure 4D). The CAF-1 p60 depletion also diminished the H3K56Ac restoration typically observed following UVR exposures (Figure 4E). Furthermore, the CAF-1 p60 depletion reduced H3K9Ac in a time dependent manner following UVR, but only slightly decreased the levels of H3K27Ac, H3K18Ac and H3K14Ac. These results indicated that the restoration of H3K56ac and H3K9Ac upon UVR exposure requires the CAF-1 function. Since H3K56Ac is considered a key driver for repair-driven chromatin assembly [23], the results strongly suggest that the H3K56Ac restoration is an integral part of the repair-driven CAF-1-mediated chromatin assembly operating at the sites of DNA damage repair.

**Figure 5:**
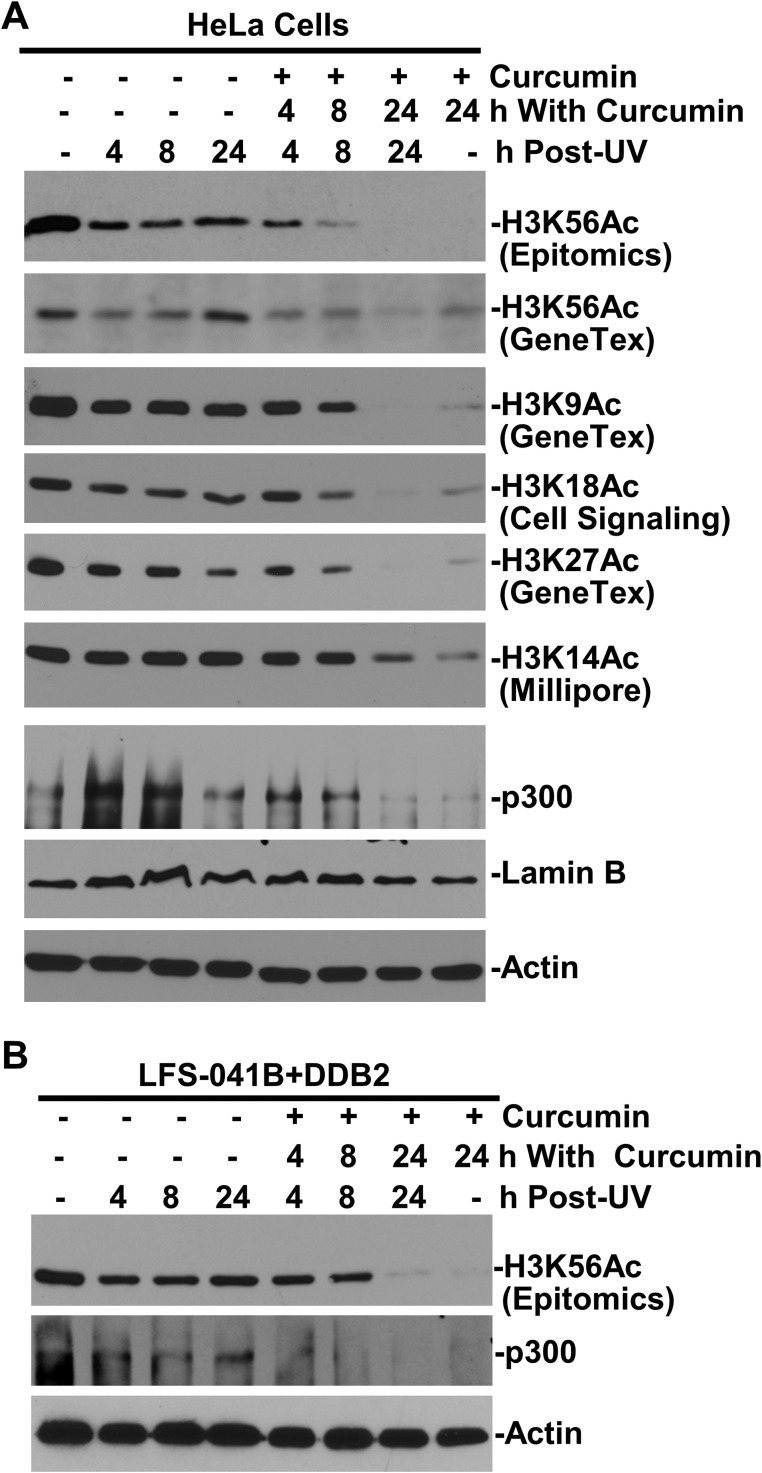
H3K56 acetylation requires CBP/p300 function (**A**) Protein levels p300, H3K56Ac and other acetylated histone H3 were examined by Western blotting in HeLa cells at indicated time periods after treatment with curcumin at 100 μM and UVR at 20 J/m^2^. (**B**) H3K56Ac and p300 levels were examined in LFS-041B+DDB2 cells after curcumin and UVR treatments. Actin blots serve as the loading controls.

To confirm that the H3K56 acetylation is mediated by histone acetyltransferase CBP/p300, we treated the cells with curcumin, which induces ubiquitin-mediated degradation of CBP/p300 [24]. The curcumin treatment diminished the cellular H3K56Ac even without any DNA damage and, not surprisingly, abolishes H3K56Ac restoration following UVR exposure (Figure 5A). Surprisingly, H3K9Ac, H3K18Ac, H3K27Ac and H3K14Ac were all diminished over 24-h period of curcumin or combined UVR and curcumin treatments. Similarly, in LFS-041B+DDB2 cells, curcumin treatment abolished the H3K56Ac restoration supported by the enforced DDB2 expression (Figure 5B). Thus, acetyltransferase p300 is functionally required for acetylations of H3 at K9, K18, K27 and K14, in addition to H3K56Ac restoration, which also requires CAF-1 function and is regulated by CRL4^DDB2^ ubiquitin ligase.

### CUL4A function is required for recruiting CAF-1 to DNA damage sites

**Figure 6:**
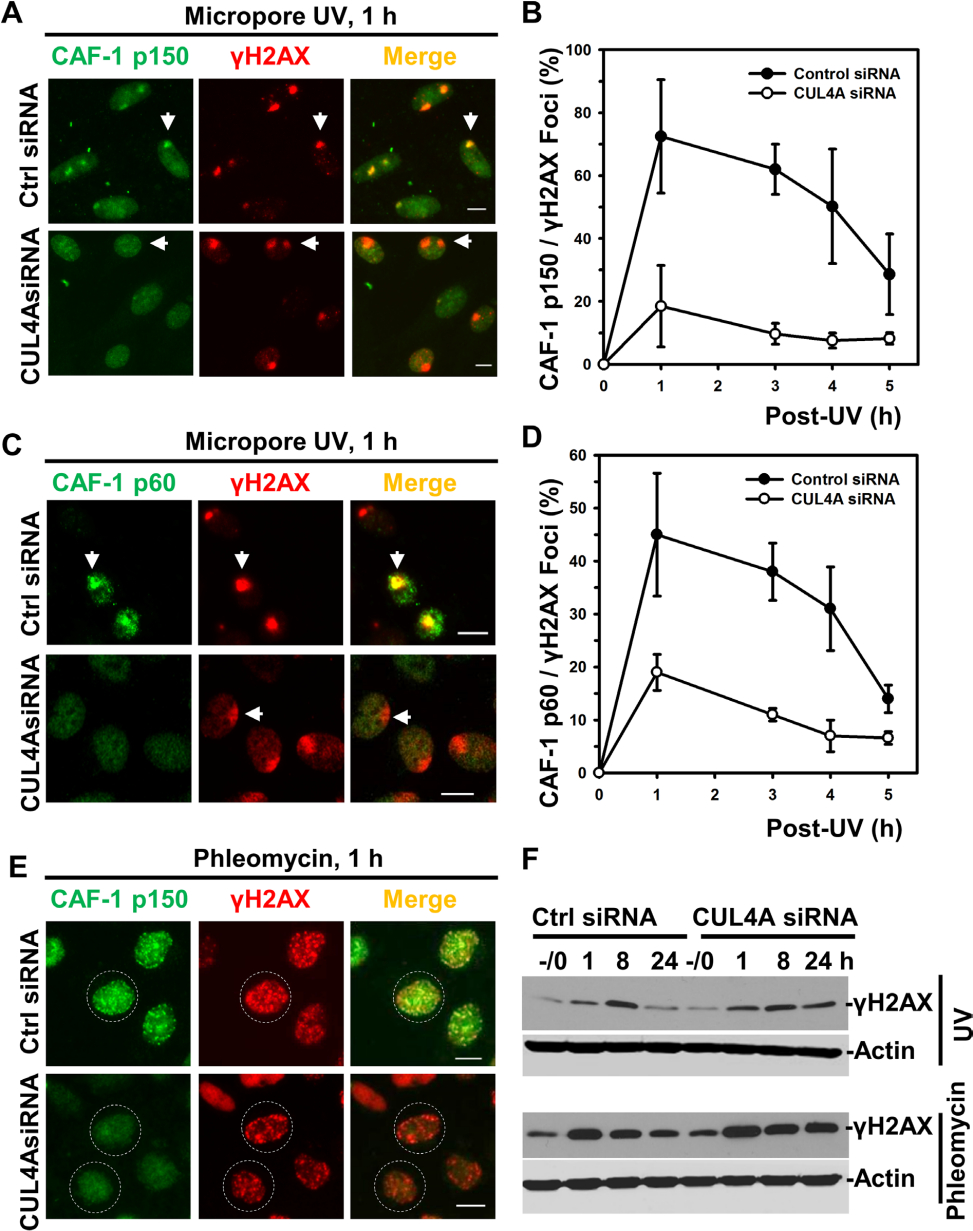
Depletion of CUL4A decreases recruitment of CAF-1 to damage sites (**A**) HeLa cells were transfected with control (Ctrl) siRNA and CUL4A siRNA. CAF-1 p150 and γH2AX were visualized by immunofluorescence in transfected cells exposed to micropore UVR. Arrows point to typical immunofluorescent focus of CAF-1 p150 or γH2AX. Calibration bar is 10 μm. (**B**) The quantitative data of CAF-1 p150 and γH2AX foci counts from multiple experiments. (**C**) the CAF-1 p60 and γH2AX were visualized by immunofluorescence. (**D**) The quantitative data of CAF-1 p60 and γH2AX foci counts from multiple experiments. (**E**) CAF-1 p150 and γH2AX were visualized by immunofluorescence in siRNA-transfected cells after phleomycin treatment. Dashed circles indicate the immunofluorescence decorated cellular area. (**F**) Western blotting analysis of γH2AX levels in control and CUL4A siRNA-transfected HeLa cells. Actin blots serve as the loading controls.

To gain insight into how CRL4^DDB2^ may regulate post-repair H3K56Ac restoration, we asked whether CUL4A depletion affects the recruitment of CAF-1 to UVR-induced DNA damage sites (Figure 6). One hour after micropore UVR exposure, CAF-1 p150 UVRIF were readily detectable in control siRNA-transfected NHFs at most DNA damage spots authenticated by concomitantly visualizing γH2AX foci (Figure 6A). The CAF-1 p150/γH2AX focus ratio declined from∼ 75% at 1 h to ∼30% at 5 h (Figure 6B). Depletion of CUL4A dramatically decreases CAF-1 p150 focus formation, cutting CAF-1 p150/γH2AX focus ratio from ∼75% to ∼20% at 1 h (Figure 6B). In addition, CAF-1 p150/γH2AX focus ratio at all the time points was significantly lower in CUL4A-depleted cells (Figure 6A and 6B). A similar pattern was seen for CAF-1 p60 in CUL4A-depleted cells (Figure 6C and 6D), although CAF-1 p60 UVRIF were detectable with lesser efficiency (Figure 6D). We also examined CAF-1 p150 focus formation upon phleomycin-induced DNA strand-breaks. The results showed that CAF-1 p150 forms multiple distinctive foci at strand-break sites following cellular phleomycin treatment (Figure 6E). CUL4A depletion renders CAF-1 p150 immunofluorescent signal diffusive, weaker and the CAF-1 p150 foci less distinguishable in most cells. Taken together, it was concluded that CUL4A function is required for recruitment of CAF-1 at DNA damage sites.

We further examined the effect of CUL4A depletion on the cellular level of γH2AX. In control siRNA-transfected NHFs, γH2AX level exhibited a sharp rise from 1 to 8 h following UVR exposure followed by a decline at 24 h. In contrast, CUL4A depletion in cells caused an increase in γH2AX level that lasted throughout the 24 h period (Figure 6F). Similarly, γH2AX level was elevated at 1 h upon phleomycin treatment and decreased over a 24-h period. Persistent γH2AX levels were also seen under CUL4A depletion with phleomycin treatment. Given that H3K56Ac signals the completion of DNA repair as well as allows the checkpoint termination, these results corroborate the functional requirement of CUL4A for H3K56Ac restoration.

### CUL4A neddylation is needed for CRL4^DDB2^ activation and recruitment of CAF-1 to DNA damage sites

**Figure 7:**
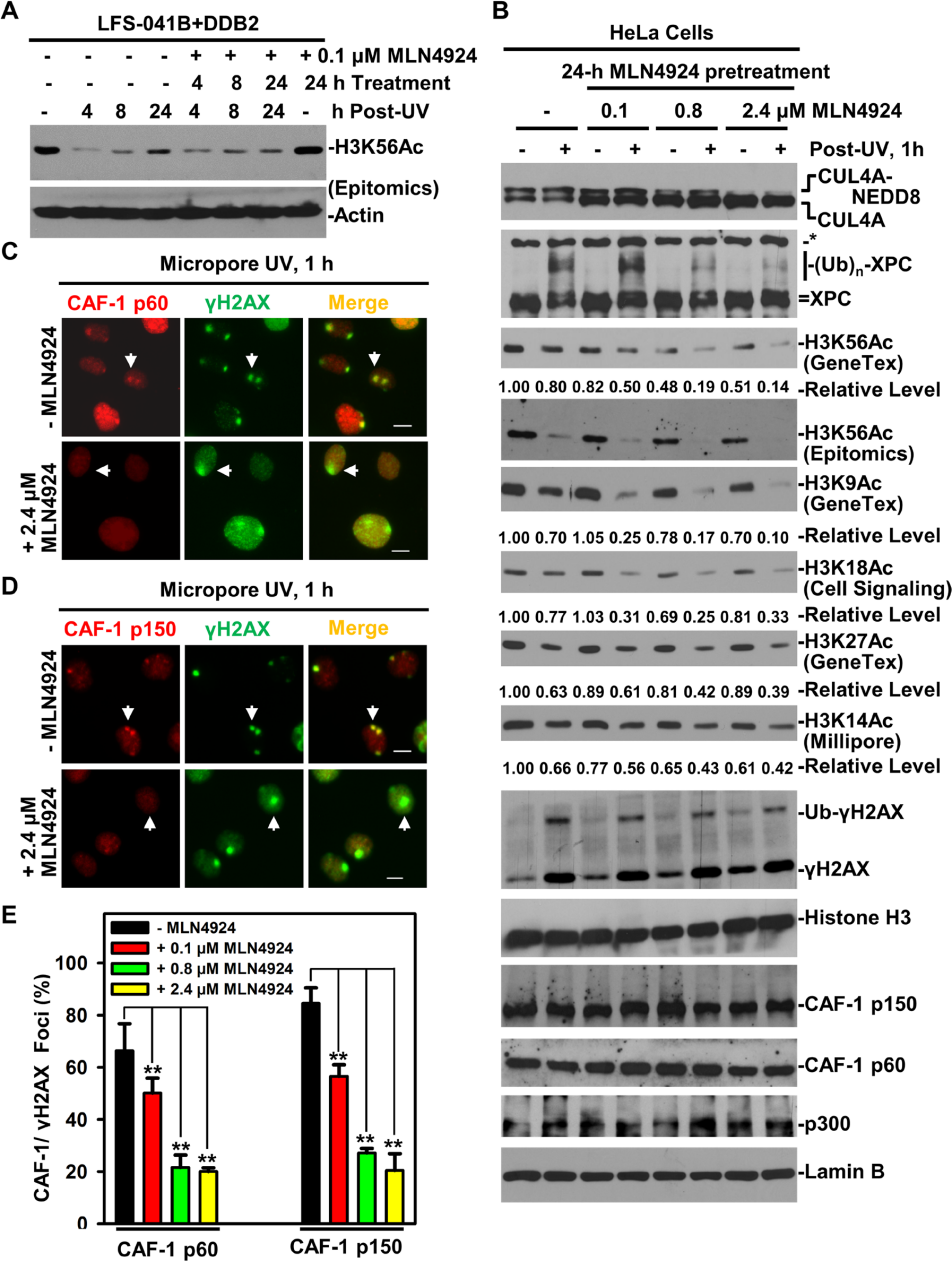
Neddylation inhibition decreases H3K56Ac and diminishes CAF-1 recruitment to damage sites (**A**) H3K56Ac were examined by Western blotting in LFS-041B+DDB2 cells at indicated time periods after treatment with neddylation inhibitor MLN4924 at 0.1 μM and UV at 20 J/m^2^. (**B**) HeLa cells were pretreated with MLN4924 at indicated concentration for 24 h, UV-irradiated at 20 J/m^2^ and were harvested 1 h thereafter. Samples of whole cell Laemmli extracts were analyzed by Western blotting for indicated proteins. Lamin B blots serve as the loading control. The blots of H3K56Ac and other acetylated histone H3 from the experiments were quantitated using ImageJ software. (**C**) CAF-1 p60 and γH2AX were visualized by immunofluorescence in HeLa cells 1 h after micropore UV irradiation at 100 J/m^2^, with or without MLN4924 treatment. (**D**) The visualization of CAF-1 p150 and γH2AX after MLN4924 treatment. Arrows point to typical immunofluorescent foci of CAF-1 p150 or γH2AX. Calibration bar is 10 μm in C and D. (**E**) The quantitative data of CAF-1 p60, p150 and γH2AX foci counted from multiple experiments in C and D. Student *t*-tests were done using Sigma Plot software. ^**^ indicates *p* ≤ 0.01.

Since activation by CUL4A neddylation is a characteristic feature of CRL4 ubiquitin ligase complex [22], we investigated whether CUL4A neddylation is involved in regulating H3K56Ac restoration. The LFS-041B+DDB2 cells were treated with neddylation inhibitor MLN4924 at 0.1 μM following UVR exposure (Figure 7A). At this MLN4924 concentration, H3K56Ac return levels at 24 h were reduced by MLN4924, while MLN4924 alone did not affect H3K56Ac level without UVR. Thus, the H3K56Ac return levels normally observed upon UVR exposures are sensitive to neddylation inhibition.

We further pretreated HeLa cells for 24 h with MLN4924 at variable concentration and examined the effect at 1 h following UVR exposure (Figure 7B). As expected, CUL4A neddylation, as indicated by the presence of NEDD8-conjugated CUL4A, was inhibited by MLN4924 in a dose-dependent manner. Meanwhile, the CRL4^DDB2^-mediated XPC ubiquitination was blocked by 0.8 and 2.4 μM of MLN4924. More importantly, H3K56Ac level decreased in a MLN4924-dose-dependent manner, reaching ∼50% at 2.4 μM and decreased further to ∼14% by applying UVR. Similarly, H3K9Ac level was reduced by MLN4924 albeit to lesser extent but further reduced to ∼10% by UVR. On the other hand, the MLN4924 pretreatment decreased H3K18Ac, H3K27Ac and H3K14Ac levels, which were all further reduced by UVR to ∼33%, ∼39% and ∼42%, respectively. Thus, H3K56Ac and H3K9Ac are preferentially downregulated by the neddylation inhibitor. Further, the neddylation inhibition increased the γH2AX without UVR while reduced the mono-ubiquitinated γH2AX level upon UVR. By contrast, the neddylation inhibition did not affect the cellular levels of Histone H3, CAF-1 p150, p60 or histone acetyltransferase p300. Thus, CUL4A neddylation is required for maintaining H3K56Ac and H3K9Ac levels to a comparatively greater extent than H3K18Ac, H3K27Ac and H3K14Ac levels.

We next explored whether the neddylation inhibition affects the recruitment of CAF-1 to UVR-induced DNA damage sites. HeLa cells were similarly pretreated with MLN4924 at variable concentrations for 24 h before delivering UVR *via* micropore filters. Without MLN4924 treatment, CAF-1 p60 formed UVRIF on DNA damage spots (Figure 7C) at a 65% CAF-1 p60/γH2AX focus ratio (Figure 7E), while CAF-1 p150 formed UVRIF (Figure 7D) at 80% CAF-1 p150/γH2AX focus ratio (Figure 7E). On the contrary, the formation of CAF-1 p60 and p150 UVRIF was blocked by MLN4924 in a dose-dependent manner (Figure 7C–7E). For example, at 0.1 μM of MLN4924, CAF-1 p60/γH2AX focus ratio decreased from ∼65% to 50%. Likewise, CAF-1 p150/γH2AX focus ratio declined from ∼80% to 55%. At higher MLN4924 concentrations (0.8 and 2.4 μM), both CAF-1 p60/γH2AX and CAF-1 p150/γH2AX focus ratio fell to ∼20%, suggesting that neddylation inhibition effectively eradicates both CAF-1 p60 and p150 UVRIF. We concluded that neddylation-mediated activation of CRL4 ubiquitin ligase activity is required for the recruitment or retention of CAF-1 p60 and p150 to DNA damage repair sites.

### CRL4^DDB2^ interacts with PCNA through DDB2 to promote H3K56 acetylation

**Figure 8:**
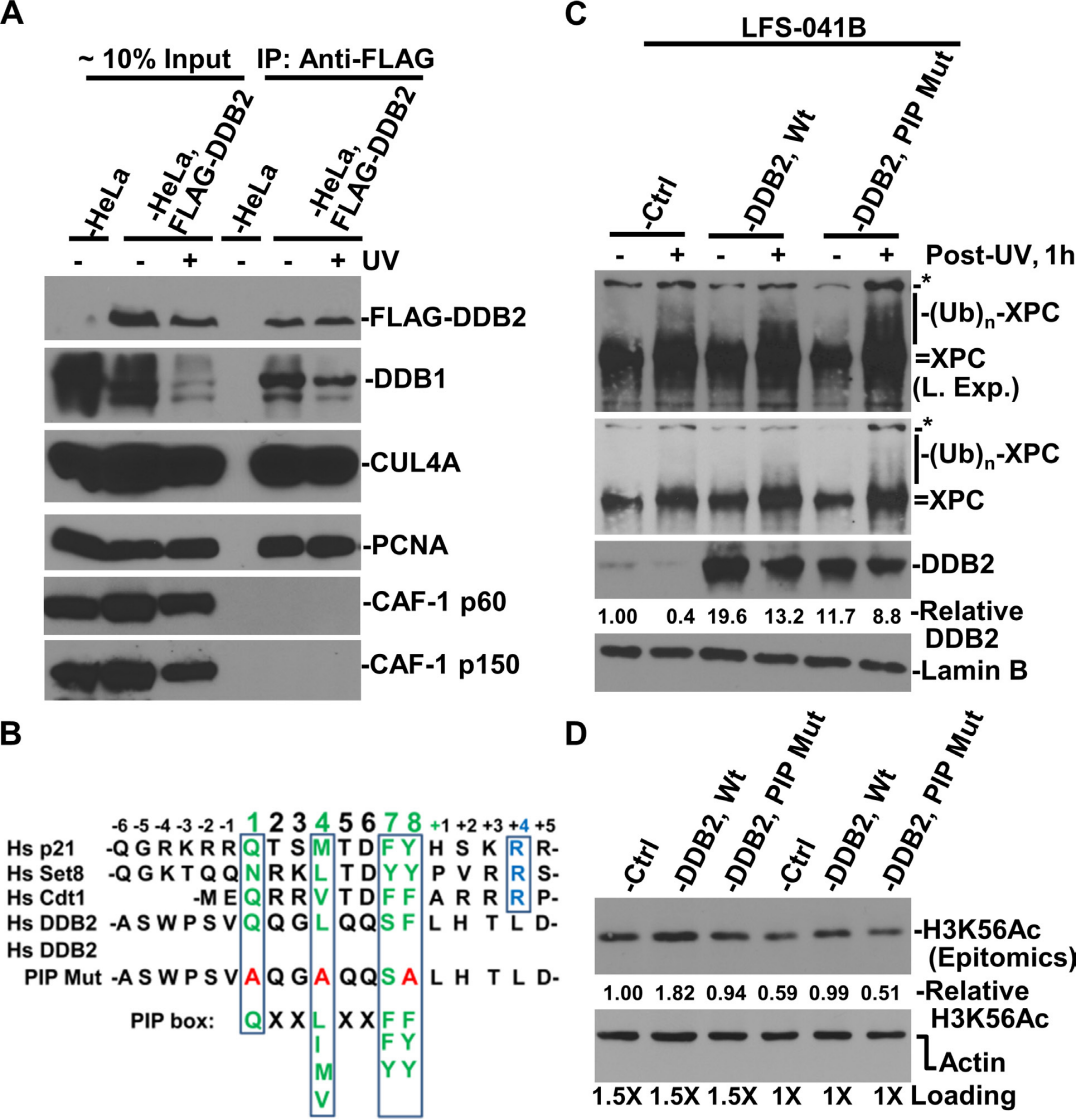
Interaction between DDB2 with PCNA is in part responsible for maintaining H3K56Ac level (**A**) Immunoprecipitation experiments were performed using soluble chromatin fractions made from parental HeLa and stable HeLa-DDB2 cells. The immunoprecipitates were analyzed by Western blotting for the presence of FLAG (DDB2), DDB1, PCNA, CUL4A and CAF-1 p60 and p150. (**B**) Alignment of PIP box and nearby sequences. Green letters highlight conserved amino acids; blue letters indicate additional conserved amino acids of PIP degron; red letters indicate amino acids in mutated PIP box of DDB2. (**C**) Mutation in DDB2 PIP box did not affect XPC ubiquitination. XPC protein and its modified species were examined in control and DDB2 vector-transfected and UV-irradiated LFS-41B cells. (**D**) H3K56Ac and actin levels were examined in control and DDB2 vector-transfected LFS-41B cells and quantitated using ImageJ software. The relative H3K56Ac level is normalized against the actin control.

To explore a possibility that CRL4^DDB2^ directly interacts with CAF-1 complex, we isolated soluble chromatin fraction from HeLa cells harboring FLAG-tagged DDB2 for immunoprecipitation. The results showed that PCNA, rather than CAF-1 p60 or p150, was present in anti-DDB2 immunoprecipitates regardless of DNA damage (Figure. 8A). This is surprising, as CUL4A function was seen to be essential for efficient recruitment of both CAF-1 p60 and p150 to damage sites (Figure 7). However, the results appear to be consistent with the finding that DDB2 interacts with PCNA *via* a PCNA-interacting protein motif, PIP box [39].

In a previous study [39], a PIP box was identified within amino acids 87 to 94 of N-terminus of human DDB2 (Figure 8B). Thus, we tested whether DDB2 PIP mutation would affect CRL4 ubiquitin ligase activity towards XPC (Figure 8C). The results showed that as compared with control, the XPC ubiquitination is enhanced by transient expression of Wild-type (Wt) DDB2. On the other hand, the PIP-mutant DDB2, despite its expression at slightly lower level, enables CRL4 to better ubiquitinate XPC, as is indicated by the presence of ubiquitin-conjugated XPC species. Thus, DDB2 with a mutated PIP box can still function as a substrate receptor of CRL4 for XPC ubiquitination. We then asked if the DDB2 PIP mutation affects H3K56 acetylation. In these experiments, we tested two loading schemes. In both cases, H3K56Ac levels are elevated by over 60% by Wt DDB2 as compared with that in LFS-041B control cells. The PIP-mutant DDB2, on the other hand, was not able to raise H3K56Ac level. Taken together, we concluded that CRL4^DDB2^ interacts with PCNA through the PIP box of DDB2 to promote H3K56 acetylation in chromatin.

## DISCUSSION

Since the discovery of a unique H3K56Ac pathway by genetic screening in budding yeast [15], the H3K56 acetylation is becoming an increasingly important phenomenon in unraveling the mechanisms of chromatin dynamics in various cellular processes, e.g., transcription, DNA replication and DNA damage response. In this study, we tested the hypothesis that CRL4^DDB2^ ubiquitin ligase regulates post-repair chromatin restoration of H3K56Ac and provide several lines of evidence in support of such a regulation. First, CRL4^DDB2^ E3 ubiquitin ligase is preferentially required for maintaining the levels of H3K56Ac and for post-repair chromatin restoration of H3K56Ac. In our experiments, DDB2 is required for maintaining the levels of H3K56Ac and H3K9Ac, but has a much lesser effect on H3K27Ac, H3K18Ac and H3K14Ac marks. Other CRL4^DDB2^ components, i.e., DDB1 and Cul4A, are also required for maintaining H3K56Ac level. In the case of UVR-induced DNA photolesions, H3K56Ac but not H3K9Ac, H3K27Ac, H3K18Ac and H3K14Ac exhibits a robust biphasic post-UV response, and the restoration of H3K56Ac requires the function of DDB2 and CUL4A. In the case of phleomycin-induced strand breaks, H3K56Ac and H3K9Ac exhibits typical biphasic response in DDB2-expressing cells, while H3K27Ac and H3K18Ac and H3K14Ac exhibit only a very weak biphasic response. When cells are deficient in DDB2, the H3K56Ac restoration is severely compromised while H3K9Ac, H3K14Ac, H3K18Ac and H3K27Ac restoration is affected to a much lesser extent. Moreover, the post-damage preferential regulation of H3K56Ac restoration by CRL4^DDB2^ E3 ligase also involves the participation by CAF-1. In our experiments (Figure 4), the ablation of CAF-1 p60 function abolished the restoration of H3K56Ac and H3K9Ac but not H3K27Ac, H3K18Ac and H3K14Ac restoration. These results are consistent with the active role of CAF-1 in post-repair chromatin assembly [8–11] and the role of H3K56Ac in driving repair-dependent chromatin assembly [23]. The results also imply that H3K56Ac and H3K9Ac may be partially co-regulated in the process. We also provide evidence for the functional requirement of CRL4^DDB2^ for the recruitment of histone chaperon CAF-1 to damage sites of UVR-induced photolesions and phleomycin-induced strand-breaks. In our experiments, knockdown of CUL4A severely compromised the recruitment of CAF-1 p150 and p60 to UV-induced damage spots, suggesting that the regulation of post-repair H3K56Ac restoration is ultimately mediated through monitoring of CAF-1 recruitment to DNA damage sites. Towards the collaborative role of CRL4^DDB2^ in regulating CAF-1 recruitment, we found that inhibition of CUL4A neddylation eliminates the recruitment of histone chaperon CAF-1 to damage sites. Finally, our experiments showed that the PIP box within N-terminal of DDB2 was essential for maintaining the genomic H3K56Ac levels. The aforementioned reasoning strongly argue that CRL4^DDB2^ preferentially regulates post-repair H3K56Ac restoration through modulation of CAF-1 recruitment. These combined results, alongside the previous findings that CRL4^DDB2^ ubiquitinates histones H3, H4, H2A and H2B [34] and that CUL4A and DDB1 regulate deposition of new H3.1 and H3.3 in human cells [20, 40], further suggest that CRL4^DDB2^ is the key CRL4, which essentially regulates the crosstalk between histone acetylation and ubiquitination during chromatin assembly [20].

H3K56Ac is an abundant histone modification in yeast, accounting for ∼17% of H3 histone molecules [41]. The H3K56Ac level in mammalian cells, however, is significantly low at ∼1% [24] or even lower (∼0.04%) by some reports [42]. It is conceivable that incorporation of H3K56Ac into chromatin is more restricted in mammalian cells than in yeast. The mechanism for such a difference, however, is currently unknown. In our experiments, siRNA-mediated depletion of CRL4^DDB2^ components DDB2, DDB1 and CUL4A decreases H3K56Ac level in both post-repair cells as well as undamaged cells. This phenomenon is different from H3K56Ac in yeast where cells lacking Rtt101, Mms1 or Mms22 have normal levels of H3K56Ac [15]. This is understandable because H3K56 acetylation in yeast can be carried out on unchromatinized H3-H4, by Asf1-Rtt109 functional modules. Further, it may be inferred that the H3K56 acetylation, which also requires Asf1 function in mammalian cells [24, 43], is more tightly linked to CRL4-mediated ubiquitination than that in yeast. Indeed, several protein-protein interactions may account for the potential mechanisms. For example, interaction between histone acetyltransferase p300 and PCNA [44, 45], and between p300 and DDB2/DDB1 [46, 47] have been identified. In particular, p300 associates with repair-synthesized DNA [44]. In line with these observations, we found that DDB2 interacts with PCNA through a PIP box, and that such an interaction is required for DDB2 to stimulate H3K56 acetylation (Figure 8). PCNA is therefore likely to act as a hub for bringing p300, CRL4^DDB2^, CAF-1 and other components in close physical proximity to newly synthesized DNA for repair-driven chromatin assembly.

Our studies revealed that CAF-1 recruitment to DNA damage sites depends on CRL4^DDB2^ function (Figures 6 and 7). This regulation somewhat resembles the regulation of H3.3 chaperon HIRA, which is responsible for repair- and replication-independent deposition of histone H3.3 at UVR-induced DNA damage sites [40]. Like CAF-1, H3.3 chaperon HIRA complex is recruited to damage sites in CRL4^DDB2^-dependent manner. However, the CAF-1, but not HIRA recruitment, require functional NER [9, 40]. Notably, HIRA also mediates the incorporation of H3.3K56Ac at chromatin *via* a histone 3 exchange mechanism [48]. Thus, it is possible that CRL4^DDB2^ regulates both recruitment of CAF-1 and HIRA through common or similar substrate(s) and it is tempting to speculate that histone H3.1, H3.3 and H4 are functionally relevant substrates. In case of HIRA-mediated H3.3 deposition, ubiquitination of H3 and H4 may result in histone exchanges at DNA damage sites. In parallel, H3 and H4 ubiquitination may lead to H3 and H4 deposition for repair-driven nucleosome assembly. Other potential substrates for CRL4^DDB2^ that could be explored in future studies are CBP/p300 and PCNA. The ubiquitin modification may regulate enzymatic activity or physical properties of these proteins to enhance chromatinization of newly synthesized DNA.

## MATERIALS AND METHODS

### Cell cultures, transfection and RNA interference

Li-Fraumeni syndrome (LFS) fibroblast 041B cell line (LFS-041B) was provided by Dr. Michael Tainsky (M.D. Anderson Cancer Center, Houston, TX 77030). LFS-041B lacks DDB2 expression due to p53-Null status. Normal human fibroblasts (NHFs) and LFS-041+DDB2 cells, which stably express V5-His-tagged DDB2, were established in our laboratory [49]. HeLa-DDB2.com cells, expressing HA- and FLAG-tagged DDB2, were kindly provided by Dr. Yoshihiro Nakatani (Dana-Farber Cancer Institute, Boston, MA 02215). NHFs, LFS-041, HeLa and HeLa-DDB2.com cells were cultured in Dulbecco’s Modified Eagle’s Medium (DMEM) supplemented with 10% fetal bovine serum (FBS). All cells were grown at 37°C in a humidified atmosphere with 5% CO_2_.

All small interfering RNA (siRNA) oligonucleotides were synthesized by Dharmacon (Lafayette, CO 80026) in a purified and annealed duplex form. The targeting sequences are siDDB2–1: 5′-CAA CUA GGC UGC AAG ACU U-3′; siDDB2–2: 5′-GAU AUC AUG CUC UGG AAU U-3′; siDDB1: 5′-UAA CAU GAG AAC UCU UGU C-3′; siCUL4A: 5′-GAA CAG CGA UCG UAA UCA AUU-3′; and control (Ctrl) siRNA: 5′-UUC UCC GAA CGU GUC ACG UdTdT-3′. The siRNA transfection was conducted using Lipofectamine 2000 reagents (Life Technologies, Grand Island, NY 14072), as described earlier [50, 51].

### Antibodies, chemicals and DNA constructs

Antibodies against H3K56Ac, H3K9Ac and H3K27Ac were obtained from Epitomics-Abcam and GeneTex (Irvine, CA 92606). H3K14Ac, H3K18Ac and γ-H2AX antibodies were from Millipore (Billerica, MA 01821, and Cell Signaling (Danvers, MA 01923). Anti-XPC antibody (XPC-2) was generated by immunizing rabbits with a synthetic peptide corresponding to the C-terminus of human XPC protein [52]. Anti-FLAG M2 agarose beads and anti-FLAG M2 antibody were purchased from Sigma-Aldrich (St. Louis, MO 63103). CUL4A (A300-739A), p300 (A300-358A) and DDB1 (A300-462A) antibodies were from Bethyl Laboratories. Anti-DDB2 (AF3297) was purchased from R&D Systems (Minneapolis, MN 55413); CAF-1 p60 (Ab8133), p150 (Ab7655) and CAF-1 p60 (K0116-3), p150 (K0115-3) were obtained from Abcam and MBL International Corporation (Woburn, MA 01801). Biochemical phleomycin was purchased from Sigma-Aldrich, and, Nedd8-activating enzyme inhibitor MLN4924 from Active Biochem (Maplewood, NJ 07040).

DDB2 expression construct expressing wild type (Wt) DDB2 was constructed earlier in our laboratory [49]. DDB2 PIP mutant construct [39] was provided by Dr. Ornella Cazzalini (Department of Molecular Medicine, University of Pavia, Pavia 27100, Italy).

### Micropore UV irradiation and immunofluorescence

HeLa cells or siRNA-transfected or MLN2924-treated HeLa cells were grown on glass coverslips at a desired density. Micropore UV radiation was conducted by placing a 5-μm isopore polycarbonate filter (EMB Millipore, Billerica, MA 01821) on the cells, followed by UV irradiation at indicated doses [53]. The UV-irradiated cells were maintained in a suitable medium for indicated times. For immunofluorescent staining, the cells were washed twice with cold PBS, treated with pre-extraction buffer (25 mM HEPES, PH 7.5, 50 mM NaCl, 1 mM EDTA, 3 mM MgCl_2_, 300 mM sucrose, 0.5% Triton X) for 2.5 min at 4°C as needed, and/or fixed with 2% paraformaldehyde in 0.5% Triton X-100 at 4°C for 30 min. The fixed cells were rinsed twice with cold PBS, blocked with 20% normal goat serum, and stained with an appropriate primary antibody as well as fluorescein isothiocyanate (FITC) or Alexa Fluor 488 or Texas Red-conjugated secondary antibodies. The coverslips were mounted in Vectashield mounting medium with DAPI. The fluorescence images were obtained with a Nikon fluorescence microscope E80i (Tokyo, Japan) and processed with SPOT and ImageJ software.

### Cellular protein fractionation and immunoprecipitation

The experiments were conducted as described by Anindya et al. [54], with modifications. Briefly, cells (∼10^7^) were lysed with 1 ml (∼5x cell volume) of cytoplasmic lysis buffer (10 mM Tris-HCl [pH 7.9], 0.34 M sucrose, 3 mM CaCl_2_, 2 mM magnesium acetate, 0.1 mM EDTA, 1 mM DDT, 0.5% NP-40 and a protease inhibitor cocktail). Nuclei were pelleted by centrifugation at 3,500 xg for 15 min and washed with cytoplasmic lysis buffer without NP-40 and then lysed in 1 ml of nuclear lysis buffer (20 mM HEPES [pH 7.9], 3 mM EDTA, 10% glycerol, 1.5 mM MgCl_2_, 150 mM KOAc and protease inhibitors). The nucleoplasmic fractions were separated by centrifugation at 15,000 g for 30 min and the pellets were designated as chromatin fraction. For further processing, the pellets were resuspended in 0.2 ml of nuclease incubation buffer (150 mM HEPES [pH 7.9], 1.5 mM MgCl_2_, 150 mM KOAc and protease inhibitors) and incubated with 50 U Benzonase (25 U/μl) for 30 min at room temperature. The soluble chromatin fraction was collected by centrifugation at 20,000 xg for 30 min, while the insoluble chromatin fraction was dissolved by boiling in SDS sample buffer.

The immunoprecipitation was done at 4°C overnight in RIPA buffer (50 mM Tris-HCl [pH 8.0], 150 mM NaCl, 1% NP40, 0.5% deoxycholate and protease inhibitors) using nuclease-releasable chromatin containing ∼500-1000 μg protein and the anti-FLAG-M2 beads. The beads were washed 1x with RIPA buffer and then 3x with TBS buffer (50 mM Tris-HCl [pH 7.4] 150 mM NaCl) and the bound proteins were eluted with FLAG peptide as described in the manufacturer’s protocol.

### Quantitative analysis and statistics

Quantitative analysis was done on digitalized Western blotting images and immunofluorescence images by ImageJ software. Student’s *t*-test was performed using SigmaPlot software. Quantitative analysis was done on digitalized Western blotting images and immunofluorescence images by ImageJ software. Student’s *t*-test was performed using SigmaPlot software.

## Abbreviations

H3K56Ac: acetylated histone H3 lysine 56
DDB1: damaged DNA binding protein 1
DDB2: damaged DNA binding protein 2
CAF-1: chromatin assembly factor-1
PCNA: proliferation cell nuclear antigen
Asf1: anti-silencing function 1
DCAF: DDB1- and CUL4A-associated factor
PTM: post-translational histone modification
UVR: ultraviolet radiation
IR: ionizing radiation
HDAC: histone deacetylase
NER: nucleotide excision repair
γH2AX: phosphorylated histone H2A variant
CRL4: DDB1-CUL4-Rbx1 ubiquitin ligase
UVRIF: ultraviolet radiation induced fluorescent foci

## References

[1] Kornberg RD (1977). Structure of chromatin. Annu Rev Biochem.

[2] Polo SE, Almouzni G (2015). Chromatin dynamics after DNA damage: The legacy of the access-repair-restore model. DNA Repair (Amst).

[3] Kaufman PD, Kobayashi R, Kessler N, Stillman B (1995). The p150 and p60 subunits of chromatin assembly factor I: a molecular link between newly synthesized histones and DNA replication. Cell.

[4] Verreault A, Kaufman PD, Kobayashi R, Stillman B (1996). Nucleosome assembly by a complex of CAF-1 and acetylated histones H3/H4. Cell.

[5] Shibahara K, Stillman B (1999). Replication-dependent marking of DNA by PCNA facilitates CAF-1-coupled inheritance of chromatin. Cell.

[6] Rolef Ben-Shahar T, Castillo AG, Osborne MJ, Borden KL, Kornblatt J, Verreault A (2009). Two fundamentally distinct PCNA interaction peptides contribute to chromatin assembly factor 1 function. Mol Cell Biol.

[7] Tagami H, Ray-Gallet D, Almouzni G, Nakatani Y (2004). Histone H3.1 and H3.3 complexes mediate nucleosome assembly pathways dependent or independent of DNA synthesis. Cell.

[8] Gaillard PH, Martini EMD, Kaufman PD, Stillman B, Moustacchi E, Almouzni G (1996). Chromatin assembly coupled to DNA repair: A new role for chromatin assembly factor I. Cell.

[9] Green CM, Almouzni G (2003). Local action of the chromatin assembly factor CAF-1 at sites of nucleotide excision repair *in vivo*. EMBO J.

[10] Polo SE, Roche D, Almouzni G (2006). New histone incorporation marks sites of UV repair in human cells. Cell.

[11] Moggs JG, Grandi P, Quivy JP, Jonsson ZO, Hubscher U, Becker PB, Almouzni G (2000). A CAF-1-PCNA-mediated chromatin assembly pathway triggered by sensing DNA damage. Mol Cell Biol.

[12] Campos EI, Smits AH, Kang YH, Landry S, Escobar TM, Nayak S, Ueberheide BM, Durocher D, Vermeulen M, Hurwitz J, Reinberg D (2015). Analysis of the Histone H3.1 Interactome: A Suitable Chaperone for the Right Event. Mol Cell.

[13] Campos EI, Fillingham J, Li G, Zheng H, Voigt P, Kuo WH, Seepany H, Gao Z, Day LA, Greenblatt JF, Reinberg D (2010). The program for processing newly synthesized histones H3.1 and H4. Nat Struct Mol Biol.

[14] Mello JA, Sillje HH, Roche DM, Kirschner DB, Nigg EA, Almouzni G (2002). Human Asf1 and CAF-1 interact and synergize in a repair-coupled nucleosome assembly pathway. EMBO Rep.

[15] Collins SR, Miller KM, Maas NL, Roguev A, Fillingham J, Chu CS, Schuldiner M, Gebbia M, Recht J, Shales M, Ding H, Xu H, Han J (2007). Functional dissection of protein complexes involved in yeast chromosome biology using a genetic interaction map. Nature.

[16] Driscoll R, Hudson A, Jackson SP (2007). Yeast Rtt109 promotes genome stability by acetylating histone H3 on lysine 56. Science.

[17] Han J, Zhou H, Horazdovsky B, Zhang K, Xu RM, Zhang Z (2007). Rtt109 acetylates histone H3 lysine 56 and functions in DNA replication. Science.

[18] Schneider J, Bajwa P, Johnson FC, Bhaumik SR, Shilatifard A (2006). Rtt109 is required for proper H3K56 acetylation: a chromatin mark associated with the elongating RNA polymerase II. J Biol Chem.

[19] Durairaj G, Chaurasia P, Lahudkar S, Malik S, Shukla A, Bhaumik SR (2010). Regulation of chromatin assembly/disassembly by Rtt109p, a histone H3 Lys56-specific acetyltransferase, *in vivo*. J Biol Chem.

[20] Han J, Zhang H, Zhang H, Wang Z, Zhou H, Zhang Z (2013). A Cul4 E3 ubiquitin ligase regulates histone hand-off during nucleosome assembly. Cell.

[21] Zaidi IW, Rabut G, Poveda A, Scheel H, Malmstrom J, Ulrich H, Hofmann K, Pasero P, Peter M, Luke B (2008). Rtt101 and Mms1 in budding yeast form a CUL4(DDB1)-like ubiquitin ligase that promotes replication through damaged DNA. EMBO Rep.

[22] Jackson S, Xiong Y (2009). CRL4s: the CUL4-RING E3 ubiquitin ligases. Trends Biochem Sci.

[23] Chen CC, Carson JJ, Feser J, Tamburini B, Zabaronick S, Linger J, Tyler JK (2008). Acetylated lysine 56 on histone H3 drives chromatin assembly after repair and signals for the completion of repair. Cell.

[24] Das C, Lucia MS, Hansen KC, Tyler JK (2009). CBP/p300-mediated acetylation of histone H3 on lysine 56. Nature.

[25] Vempati RK, Jayani RS, Notani D, Sengupta A, Galande S, Haldar D (2010). p300-mediated acetylation of histone H3 lysine 56 functions in DNA damage response in mammals. J Biol Chem.

[26] Tjeertes JV, Miller KM, Jackson SP (2009). Screen for DNA-damage-responsive histone modifications identifies H3K9Ac and H3K56Ac in human cells. EMBO J.

[27] Miller KM, Tjeertes JV, Coates J, Legube G, Polo SE, Britton S, Jackson SP (2010). Human HDAC1 and HDAC2 function in the DNA-damage response to promote DNA nonhomologous end-joining. Nat Struct Mol Biol.

[28] Battu A, Ray A, Wani AA (2011). ASF1A and ATM regulate H3K56-mediated cell-cycle checkpoint recovery in response to UV irradiation. Nucleic Acids Res.

[29] Zhu Q, Battu A, Ray A, Wani G, Qian J, He J, Wang QE, Wani AA (2015). Damaged DNA-binding protein down-regulates epigenetic mark H3K56Ac through histone deacetylase 1 and 2. Mutat Res.

[30] Chu G, Chang E (1988). Xeroderma pigmentosum group E cells lack a nuclear factor that binds to damaged DNA. Science.

[31] Groisman R, Polanowska J, Kuraoka I, Sawada J, Saijo M, Drapkin R, Kisselev AF, Tanaka K, Nakatani Y (2003). The ubiquitin ligase activity in the DDB2 and CSA complexes is differentially regulated by the COP9 signalosome in response to DNA damage. Cell.

[32] Sugasawa K (2010). Regulation of damage recognition in mammalian global genomic nucleotide excision repair. Mut Res.

[33] Scrima A, Fischer ES, Lingaraju GM, Bohm K, Cavadini S, Thoma NH (2011). Detecting UV-lesions in the genome: The modular CRL4 ubiquitin ligase does it best!. FEBS Lett.

[34] Wang H, Zhai L, Xu J, Joo HY, Jackson S, Erdjument-Bromage H, Tempst P, Xiong Y, Zhang Y (2006). Histone H3 and H4 ubiquitylation by the CUL4-DDB-ROC1 ubiquitin ligase facilitates cellular response to DNA damage. Mol Cell.

[35] Hwang BJ, Ford JM, Hanawalt PC, Chu G (1999). Expression of the p48 xeroderma pigmentosum gene is p53-dependent and is involved in global genomic repair. Proc Natl Acad Sci USA.

[36] Pal S, Graves H, Ohsawa R, Huang TH, Wang P, Harmacek L, Tyler J (2016). The Commercial Antibodies Widely Used to Measure H3 K56 Acetylation Are Non-Specific in Human and Drosophila Cells. PLoS One.

[37] El-Mahdy MA, Zhu Q, Wang QE, Wani G, Praetorius-Ibba M, Wani AA (2006). Cullin 4A-mediated proteolysis of DDB2 protein at DNA damage sites regulates *in vivo* lesion recognition by XPC. J Biol Chem.

[38] Liu L, Lee S, Zhang J, Peters SB, Hannah J, Zhang Y, Yin Y, Koff A, Ma L, Zhou P (2009). CUL4A abrogation augments DNA damage response and protection against skin carcinogenesis. Mol Cell.

[39] Cazzalini O, Perucca P, Mocchi R, Sommatis S, Prosperi E, Stivala LA (2014). DDB2 association with PCNA is required for its degradation after UV-induced DNA damage. Cell Cycle.

[40] Adam S, Polo SE, Almouzni G (2013). Transcription recovery after DNA damage requires chromatin priming by the H3.3 histone chaperone HIRA. Cell.

[41] Drogaris P, Wurtele H, Masumoto H, Verreault A, Thibault P (2008). Comprehensive profiling of histone modifications using a label-free approach and its applications in determining structure-function relationships. Anal Chem.

[42] Drogaris P, Villeneuve V, Pomies C, Lee EH, Bourdeau V, Bonneil E, Ferbeyre G, Verreault A, Thibault P (2012). Histone deacetylase inhibitors globally enhance h3/h4 tail acetylation without affecting h3 lysine 56 acetylation. Sci Rep.

[43] Das C, Roy S, Namjoshi S, Malarkey CS, Jones DN, Kutateladze TG, Churchill ME, Tyler JK (2014). Binding of the histone chaperone ASF1 to the CBP bromodomain promotes histone acetylation. Proc Natl Acad Sci U S A.

[44] Hasan S, Hassa PO, Imhof R, Hottiger MO (2001). Transcription coactivator p300 binds PCNA and may have a role in DNA repair synthesis. Nature.

[45] Cazzalini O, Sommatis S, Tillhon M, Dutto I, Bachi A, Rapp A, Nardo T, Scovassi AI, Necchi D, Cardoso MC, Stivala LA, Prosperi E (2014). CBP and p300 acetylate PCNA to link its degradation with nucleotide excision repair synthesis. Nucleic Acids Res.

[46] Rapic-Otrin V, McLenigan MP, Bisi DC, Gonzalez M, Levine AS (2002). Sequential binding of UV DNA damage binding factor and degradation of the p48 subunit as early events after UV irradiation. Nucleic Acids Res.

[47] Datta A, Bagchi S, Nag A, Shiyanov P, Adami GR, Yoon T, Raychaudhuri P (2001). The p48 subunit of the damaged-DNA binding protein DDB associates with the CBP/p300 family of histone acetyltransferase. Mutat Res.

[48] Dutta D, Ray S, Home P, Saha B, Wang S, Sheibani N, Tawfik O, Cheng N, Paul S (2010). Regulation of angiogenesis by histone chaperone HIRA-mediated incorporation of lysine 56-acetylated histone H3.3 at chromatin domains of endothelial genes. J Biol Chem.

[49] Barakat BM, Wang QE, Han C, Milum K, Yin DT, Zhao Q, Wani G, Arafa el SA, El-Mahdy MA, Wani AA (2010). Overexpression of DDB2 enhances the sensitivity of human ovarian cancer cells to cisplatin by augmenting cellular apoptosis. Int J Cancer.

[50] Sharma N, Zhu Q, Wani G, He J, Wang QE, Wani AA (2014). USP3 counteracts RNF168 via deubiquitinating H2A and gammaH2AX at lysine 13 and 15. Cell Cycle.

[51] Zhu Q, Sharma N, He J, Wani G, Wani AA (2015). USP7 deubiquitinase promotes ubiquitin-dependent DNA damage signaling by stabilizing RNF168. Cell Cycle.

[52] Wang QE, Zhu Q, Wani G, El-Mahdy MA, Li J, Wani AA (2005). DNA repair factor XPC is modified by SUMO-1 and ubiquitin following UV irradiation. Nucleic Acids Res.

[53] Wang QE, Zhu Q, Wani MA, Wani G, Chen J, Wani AA (2003). Tumor supressor p53 dependent recruitment of nucleotide excision repair ractors XPC and TFIIH to DNA damage. DNA Repair.

[54] Anindya R, Aygun O, Svejstrup JQ (2007). Damage-induced ubiquitylation of human RNA polymerase II by the ubiquitin ligase Nedd4, but not Cockayne syndrome proteins or BRCA1. Mol Cell.

